# Development of *Crenosoma vulpis* in the common garden snail *Cornu aspersum*: implications for epidemiological studies

**DOI:** 10.1186/s13071-016-1483-8

**Published:** 2016-04-14

**Authors:** Vito Colella, Yasen Mutafchiev, Maria Alfonsa Cavalera, Alessio Giannelli, Riccardo Paolo Lia, Filipe Dantas-Torres, Domenico Otranto

**Affiliations:** Dipartimento di Medicina Veterinaria, Università degli Studi di Bari, Valenzano, Italy; Bulgarian Academy of Sciences, Department of Biodiversity, Institute of Biodiversity and Ecosystem Research, Sofia, Bulgaria; Department of Immunology, Aggeu Magalhães Research Centre, Oswaldo Cruz Foundation, Recife, Brazil

**Keywords:** *Crenosoma vulpis*, Metastrongyloidea, Lungworm, *Cornu aspersum*, Biology, Epidemiology, Snail, Parasite

## Abstract

**Background:**

*Crenosoma vulpis* (Dujardin, 1845), the fox lungworm, is a metastrongyloid affecting the respiratory tract of red foxes (*Vulpes vulpes*), dogs (*Canis familiaris*) and badgers (*Meles meles*) living in Europe and North America. The scant data available on the intermediate hosts of *C. vulpis*, as well as the limited information about the morphology of the larvae may jeopardise epidemiological studies on this parasite.

**Methods:**

Suitability and developmental time of *C. vulpis* in the common garden snail *Cornu aspersum* (= *Helix aspersa*) was assessed at selected days post-infection (i.e. 3, 6, 10, 15, 20 and 180). Nematodes were preserved in 70 % ethanol, cleared and examined as temporary mounts in glycerol for morphological descriptions of first- and third-stage larvae. In addition, nematodes collected from the dog and the experimentally infected snails were molecularly analysed by the amplification of the nuclear 18S rRNA gene.

**Results:**

Specimens of *C. aspersum* digested before the infection (*n* = 10) were negative for helminth infections. Out of 115 larvae recovered from infected gastropods (mean of 9.58 larvae per snail), 36 (31.3 %) were localised in the foot and 79 (68.7 %) in the viscera. The 18S rDNA sequences obtained from larvae collected from the dog and the snail tissues displayed 100 % identity to the nucleotide sequence of *C. vulpis*.

**Conclusions:**

*Cornu aspersum* is herein reported for the first time as a suitable intermediate host of *C. vulpis*. This snail species may play an important role for the infection of animals living in regions of the Mediterranean basin. In addition, this study provides more details on the morphological descriptions of L1 and L3 and supports future investigations on the epidemiology of this little known parasite.

## Background

The Superfamily Metastrongyloidea (Strongylida) includes nematode species that generally have gastropods and vertebrates as intermediate and definitive hosts, respectively [1, 2]. *Crenosoma vulpis* (Dujardin, 1845), commonly known as the fox lungworm, is a metastrongyloid affecting the respiratory tract of red foxes (*Vulpes vulpes*), dogs (*Canis familiaris*) and badgers (*Meles meles*) living in Europe and North America [3–6]. Larval stages dwell in snails and slugs, and third-stage larvae (L3) are infective to carnivore definitive hosts which feed on these gastropods [1]. Adult nematodes live in bronchioles, bronchi and trachea and produce first-stage larvae (L1) which are swallowed and passed in the faeces of infected animals in about 18–21 days post infection [1]. The infections are generally non-fatal with clinical presentations of various degrees, from subclinical to chronic respiratory syndrome, featured by lingering cough and retching [7, 8]. The better-known *Angiostrongylus vasorum* (Baillet, 1866) is another snail-borne nematode which shares some ecological and pathological features with *C. vulpis* [9]. Additionally, *A. vasorum* may also cause severe conditions due to coagulative, cardiovascular and neurological disorders in dogs [10], and its distribution seems to be increasing in previously reported and unreported geographical areas [11]. For example, *A. vasorum* has been frequently detected in Europe, Africa, North and South America [10], whereas *C. vulpis* has been diagnosed sporadically from dogs in the UK [12], Ireland [13], Switzerland [8], Germany [14], Belgium [15] and Italy [16]. For both parasites, wildlife animals are accounted as possible reservoir hosts of infection for domestic dogs [11, 17]. However, biological determinants shaping the epidemiology of *C. vulpis* have been poorly investigated. The diagnosis of lungworm infection relies on finding of L1 in the stool of infected animals by the Baermann technique [7], although a proper identification of the pathogens may be challenging due to the morphological similarities of closely related species [18]. The scant data available on the intermediate hosts of *C. vulpis*, as well as the limited information about the morphology of the larvae, may jeopardise epidemiological studies on this parasite [17]. In addition, snails and slugs experimentally demonstrated to act as intermediate hosts of *C. vulpis* (e.g. gastropods of the genera *Deroceras*, *Helix*, *Succinea*, and *Cepaea*) are mostly restricted to the northern and central regions of Europe [1]. Therefore, here we provide data on the development of *C. vulpis* in *Cornu aspersum* (= *Helix aspersa*), a common snail in regions of Mediterranean and north-western Europe, and report more details on the morphological descriptions of L1 and L3, instrumentally to support investigations on the epidemiology of this parasite.

## Methods

### Gastropod maintenance

One-year-old *Cornu aspersum* (*n* = 100) snails were purchased from a farming centre located in Barletta (Puglia, Italy). The snails were housed in a plastic box in a temperature-controlled room (21 ± 1 °C) and fed every second day with lettuce and water for two months until beginning of the study. At the arrival and one day prior to the infection, ten snails were sacrificed, digested and microscopically examined to assess the absence of any helminth infection.

### L1 collection and experimental infection of gastropods

Nematode larvae were isolated from the faeces of a 6-year-old naturally infected male Dachshund dog referred to the Parasitology Laboratory of the University of Bari (Puglia, Italy) due to respiratory distress. Faeces were collected and examined by Baermann technique for three consecutive days in order to recover nematode larvae. After the centrifugation of the faecal solution at 600 *g* for 5 min, the sediment was observed under light microscopy. The larvae obtained were morphologically and molecularly identified as *C. vulpis* (see below). The dog was successfully treated with a single application of moxidectin/imidacloprid spot on solution (Advocate^Ⓡ^, Bayer Animal Health, Leverkusen, Germany) soon after the diagnosis of canine crenosomosis.

Single infective doses of 100 L1 were collected under a light microscope (Leica^Ⓡ^, DM LB2) and utilized for infecting snails as follows. Two days unfed snails were individually placed in the infection chamber consisting of six well cell culture plate (Corning^Ⓡ^; CellBIND^Ⓡ^; Sigma-Aldrich^Ⓡ^, St. Louis, Missouri, USA) containing a potato slice (0.3 cm thick) and the infective dose. Specimens were left in the infection chambers for 48 h and subsequently returned to their box.

Suitability and developmental time of *C. vulpis* in *C. aspersum* was assessed by artificially digesting two portions (i.e. muscular foot and viscera) of two snails per time-point (i.e. 3, 6, 10, 15, 20 days post-infection, dpi). In addition, six infected snails were gradually placed at 4 °C in order to favour gastropods hibernation, replaced at laboratory condition, and analysed at 180 dpi.

Each snail was digested in 100 ml HCl solution (pH 2.2) and 3 mg/ml of pepsin (Sigma-Aldrich^Ⓡ^, St. Louis, Missouri, USA). The suspension was heated on a magnetic stirrer at 37 °C for 75 min, sifted through a 250 μm sieve to remove undigested material, transferred to 50 ml plastic tubes and centrifuged at 600 *g* for 5 min, and morphologically and molecularly analysed.

### Morphological and molecular identification of larvae

The suspension obtained from the gastropods digestion was microscopically examined and larvae were morphologically identified according to previous descriptions [19, 20]. The nematodes were preserved in 70 % ethanol and subsequently cleared and examined as temporary mounts in glycerol. Drawings were made with a compound microscope Leica DM LB2 (with differential interference contrast) equipped with a drawing tube. Digital images and measurements were taken using Leica LAS^Ⓡ^ AF 4.1 software. Metrical data are given in micrometres as the range, followed by the mean in parentheses.

For molecular identification, larval specimens of *C. vulpis* were isolated from the suspension using a 10 μl micropipette and stored in phosphate buffer saline (PBS) solution. Genomic DNA was extracted using a commercial kit (DNeasy Blood & Tissue Kit, Qiagen, GmbH, Hilden, Germany), in accordance with the manufacturer’s instructions, and a partial fragment of nuclear 18S rRNA (~1700 bp) gene was amplified as previously described [21]. The amplicons were purified and sequenced, in both directions using the same primers as for PCR, employing the Taq Dye Deoxy Terminator Cycle Sequencing Kit (v.2, Applied Biosystems, Foster City, California, USA) in an automated sequencer (ABI-PRISM 377). Sequences were compared with those available in the GenBank database, using Basic Local Alignment Search Tool (BLAST – http://blast.ncbi.nlm.nih.gov/Blast.cgi).

### Ethics statement

An ethical approval was not necessary since the present study did not involve the experimental use of vertebrates neither human patients nor protected animal species. The procedures on the infected dog were carried out with the owner’s consent.

## Results

Specimens of *C. aspersum* digested at the arrival (*n* = 5) and one day prior to the infection (*n* = 5) were negative for helminth infections. Larvae of *C. vulpis* were found in ten out of 12 (83.3 %) experimentally infected snails. The number and developmental stages of larvae detected from foot and viscera of each snail are shown in Table 1. Out of 115 larvae recovered from infected gastropods (mean of 9.58 larvae per snail), 36 (31.3 %) were localised in the foot and 79 (68.7 %) in the viscera. The 18S rDNA sequences obtained from larvae collected from the snail tissues displayed 100 % identity to the nucleotide sequence of *C. vulpis* available in GenBank (accession no. KR920038).

**Table 1:** Number of larvae and developmental stages (L1, L2 and L3) of *Crenosoma vulpis* detected by artificial digestion of foot and viscera of two *Cornu aspersum* (S1 and S2) at different days post-infection (dpi)

	Foot			Viscera			
	L1	L2	L3	L1	L2	L3	Total
S1 (3 dpi)	2	–	–	5	–	–	7
S2 (3 dpi)	–	–	–	–	–	–	0
S1 (6 dpi)	1	4	–	1	5	–	11
S2 (6 dpi)	1	2	–	1	8	–	12
S1 (10 dpi)	–	4	1	–	5	–	10
S2 (10 dpi)	–	–	–	–	–	–	0
S1 (15 dpi)	–	–	2	–	4	5	11
S2 (15 dpi)	–	–	2	–	1	9	12
S1 (20 dpi)	–	1	3	–	1	11	16
S2 (20 dpi)	–	1	4	–	1	8	14
S1 (180 dpi)	–	–	6	–	–	5	11
S2 (180 dpi)	–	–	2	–	–	9	11

### Morphological features of *C. vulpis* larvae

**Fig. 1:**
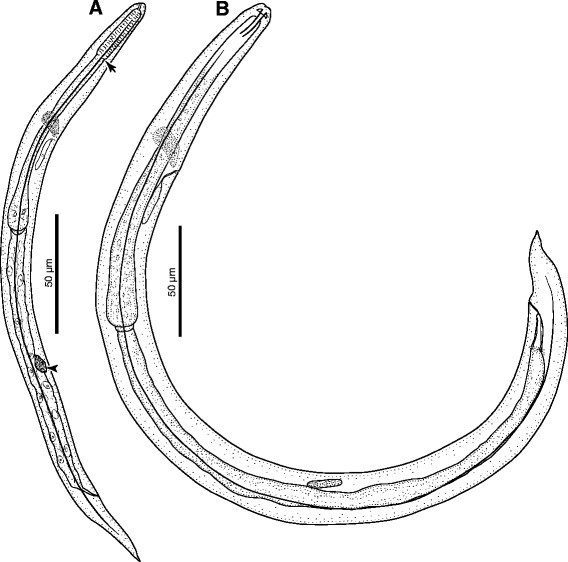
*Crenosoma vulpis*. First stage larva recovered from Baermann funnel, note the border between the procorpus and metacorpus (*arrow*) and the genital primordium (*arrowhead*) (**a**); Third stage larva at 180 days post-infection (**b**)

**Fig. 2:**
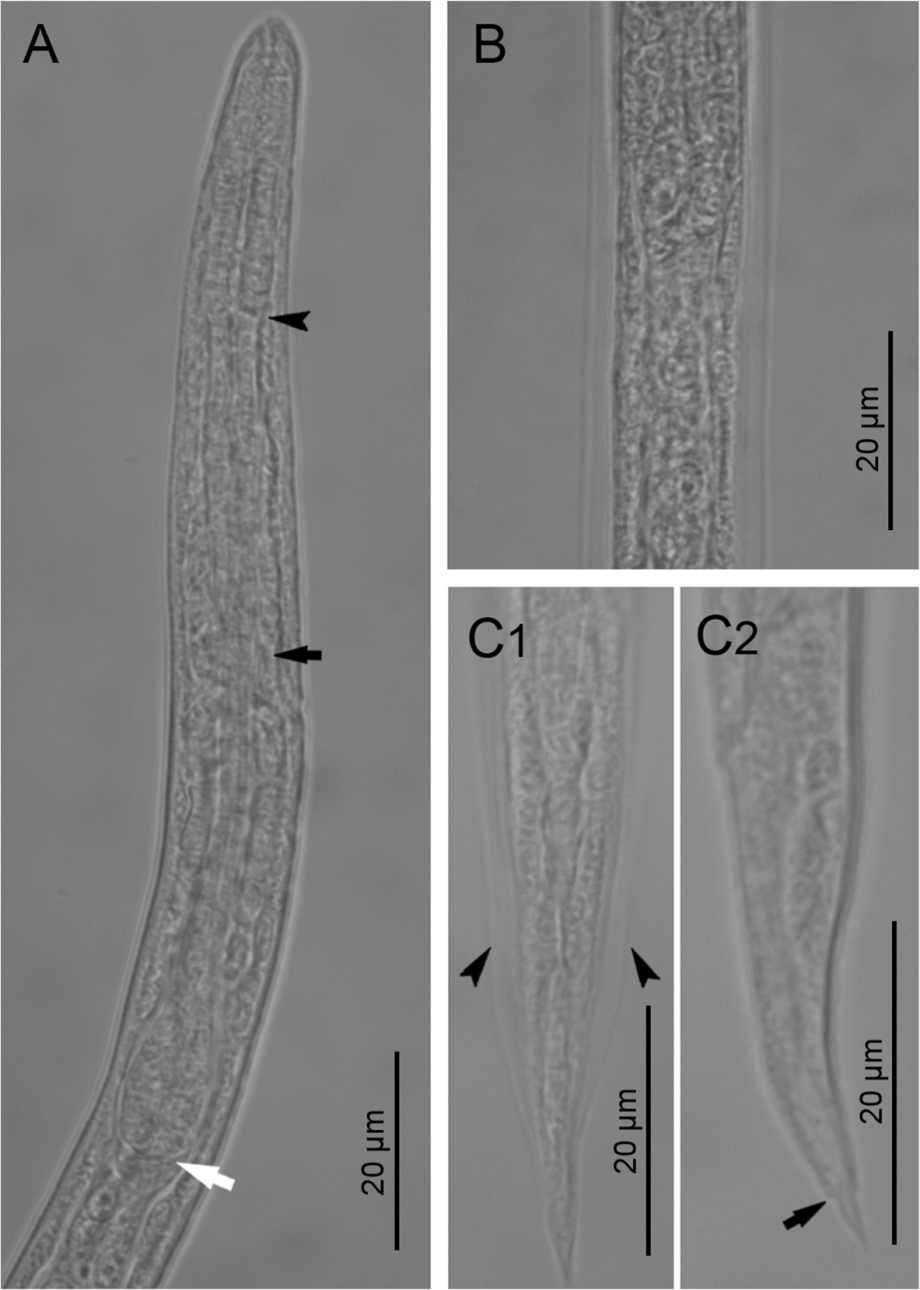
First stage larvae of *Crenosoma vulpis* recovered from Baermann funnel. Anterior extremity, lateral view, note the junction between the muscular procorpus and metacorpus (*black arrowhead*), the nerve-ring (*black arrow*) and the oesophago-intestinal junction (*white arrow*) (**a**); Lateral alae along the body at the level of the oesophago-intestinal junction, dorsoventral view (**b**); Tail, dorsoventral (C1) and lateral (C2) view; note the lateral alae (*arrowheads*) and the narrowing anterior to the pointed tip (*black arrow*) (**c**)

*First-stage larvae* (*n* = 10) filiform C-shaped, 253–280 (274) long, 12–14 wide (Fig. 1a). Body curved ventrally, lateral alae extending from head region to posterior third of tail (Fig. 2b, c). Anterior extremity triangular, with rounded apex (Figs. 1a and 2a). Tail 31–34 (33) long, straight or bent dorsally with pronounced narrowing anterior to pointed tip (Fig. 2c). Mouth opening situated dorsally to anterior apex; buccal cavity thin, *c.* 3 long. Oesophagus 99–113 (111) long, composed of cylindrical muscular procorpus 27–33 long and *c.*5 wide, followed by cylindrical metacorpus similar in length and *c.*4 wide, isthmus long and *c.*3 wide, gradually widening into pear-shaped bulb 7–8 wide (Fig. 2a). Nerve-ring at 55–62 (59) from anterior extremity. Excretory pore obscure, situated at level just posterior to nerve-ring. Genital primordium small, oval, at 156–173 (169) from anterior extremity. Ratio oesophagus length to body length 0.399–0.431 (0.407); ratio distance from anterior extremity to genital primordium to body length 0.598–0.637 (0.619).

**Fig. 3:**
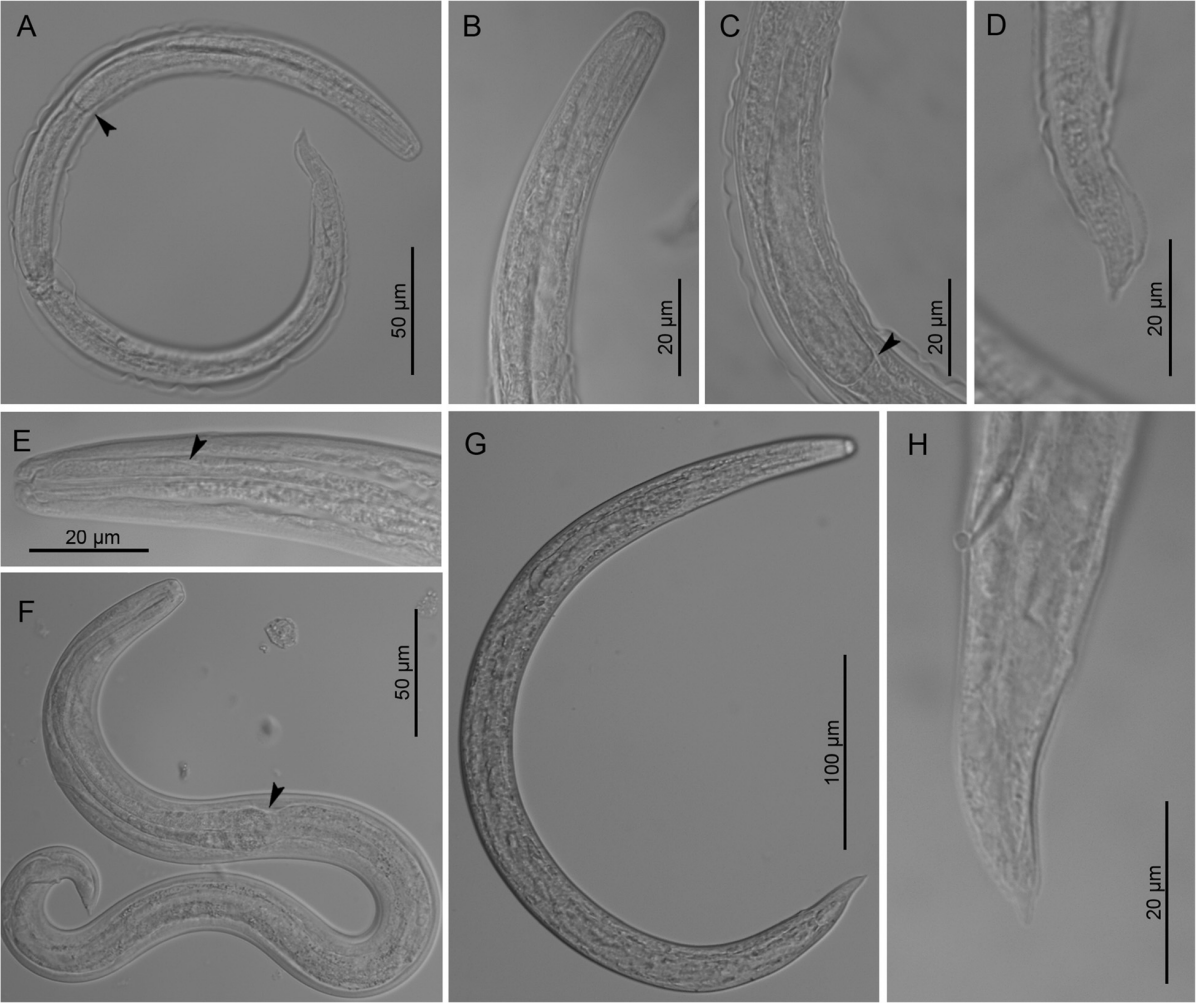
Third stage larvae of *Crenosoma vulpis* at different days post-infection (dpi), lateral view. Larva at 10 dpi, note the oesophago-intestinal junction (*arrowhead*) (**a**); Anterior extremity at 10 dpi (**b**); Posterior part of oesophagus at 10 dpi (**c**), note the oesophago-intestinal junction (*arrowhead*) and the loose cuticle of the second larval stage; Tail extremity at 10 dpi (**d**); Anterior extremity at 14 dpi (**e**), note the border between smooth and glandular parts of the oesophagus (*arrowhead*); Larva observed in vivo at 15 dpi (**f**), note the oesophago-intestinal junction (*arrowhead*); Larva at 150 dpi (**g**); Tail extremity at 180 dpi (**h**)

*Third-stage larvae* (see Table 2 for main morphological measurements). Body curved ventrally (Figs. 1b and 3a, g). Lateral alae extending from anterior body region to posterior half of tail. Mouth opening situated at blunt anterior extremity (Fig. 3b, e). Buccal cavity distinct, 10–12 long, *c*.2 wide. Oesophagus long, claviform, gradually widens posteriorly to the nerve-ring (Fig. 3a, c, f), anterior 25–30 long portion muscular followed by granular portion (Fig. 3e). Nerve-ring situated anterior to mid-length of oesophagus (Fig. 1b). Excretory pore situated just posterior to nerve-ring. Genital primordium small, oval. Tail conical, posterior half bent dorsally with distinct narrowing anterior to tail tip (Fig. 3d, h).

**Table 2:** Measurements (in micrometres) of third stage larvae of *Crenosoma vulpis* recovered from *Cornu aspersum* at selected days post-infection (dpi)

Characters/Days post-infection	10 dpi	15 dpi	20 dpi	180 dpi
Specimens studied	*n* = 1	*n* = 2	*n* = 3	*n* = 10
Body length	429	393–419	383–459 (441)	403–509 (466)
Maximum body width	16	21–22	20–22 (21)	19–23 (20)
Tail length	29	26–28	29–37 (32)	30–41 (36)
Anal body width	10	9–11	12–14 (13)	12–16 (15)
Oesophagus length	147	151–155	133–153 (134)	138–164 (152)
Oesophagus width at level of bulb	11	12–13	12–15 (13)	12–18 (14)
Genital primordium, distance from anterior extremity	268	242–259	228–293 (255)	260–303 (273)
Nerve-ring, distance from anterior extremity	70	67–70	65–67 (66)	62–88 (73)
Excretory pore, distance from anterior extremity	79	74–79	74–82 (75)	72–99 (85)
Ratio oesophagus length to body length	0.343	0.370–0.384	0.302–0.366 (0.333)	0.310–392 (0.342)
Ratio distance from anterior extremity to genital primordium to body length	0.625	0.616–0.618	0.578–0.638 (0.595)	0.575–0.639 (0.591)

## Discussion

The finding of *C. aspersum* snail as a suitable intermediate host of *C. vulpis*, together with the detailed morphological descriptions of L1 and L3, represent a step forward in the understanding of this lungworm. Although the measurements of L1 fall within the ranges previously reported for *C. vulpis* [19, 20], the pointed tail tip of this larval stage was not as elongate as reported in [20]. In addition, a complex morphology of the oesophagus has been herein described (i.e. muscular procorpus, followed by metacorpus, isthmus and basal bulb). The early L3 enclosed into the cuticle of the second larval stage (10 dpi) exhibited a similar morphology to that of L3 collected later on (i.e. 15, 20 and 180 dpi), but were distinct by the thinner body and oesophagus (see Table 2). The L3 had similar morphology of the anterior extremity, the oesophagus and the tail to those described by [19], except for their shorter body (383–509 *vs* 458–549 μm). In addition, L3 had oesophagus with a distinct anterior muscular portion followed by a longer granular portion, resembling those of other members of Metastrongyloidea, i.e. *Angiostrongylus cantonensis* (Chen, 1935), *A. vasorum* and *Aelurostrongylus abstrusus* (Railliet, 1898) [22]. Knowledge of the morphology of the larvae may be instrumental to explore the biology of *C. vulpis,* such as the L3 emergence from intermediate hosts, as already described for *A. cantonensis*, *A. vasorum*, *Troglostrongylus brevior* (Gerichter, 1949) and *A. abstrusus* [23–26]. In this study, L3 were found in the infected gastropods after six months at 4 °C, indicating that hibernated *C. aspersum* may potentially infect the definitive hosts soon after the overwintering. Therefore, in the Mediterranean regions featured by mild winter, infected snails readily available in the early spring season may cause the infection of susceptible animals by *C. vulpis*.

Recently, L3 of *A. vasorum* were detected in *C. aspersum* at 15 dpi [27], which is longer than in *C. vulpis* (i.e. 10 dpi). However, since no other time-point previous to the 15th day has been examined [27], the data are not comparable. The lack of *C. vulpis* larval development in two of the infected snails (16.6 %), as well as of *A. vasorum* (i.e. 25 % [27]) may suggest the failure in the infection procedure but also the presence of an innate immunity. For example, snail resistance to trematode infection can be promoted by innate immunity [28] or by immunological factors related to species, strain and/or age of snails [29].

The recording of a new gastropod species as intermediate host and of the morphological features of L1 and L3 of *C. vulpis* herein reported will assist and encourage future research on the biology of this little known parasite.

## Conclusions

Further investigations on the species of gastropods acting as intermediate hosts and on the genetic features of *C. vulpis* will contribute to understand the geographical distributions and risk factors associated with this parasitosis. For example, a preliminary molecular investigation of *C. vulpis* collected from foxes, dogs and one badger from Italy, revealed the existence of four haplotypes, which supported the existence of the transmission between wild and domestic carnivores [21]. However, further studies are needed to understand the role of different species of snails, slugs and vertebrate hosts in the ecology of *C. vulpis*.
